# Descriptive review of junior OB/GYN physicians’ work task financial compensation in German hospitals

**DOI:** 10.1186/s12995-019-0227-z

**Published:** 2019-03-07

**Authors:** Dörthe Brüggmann, Anja Herpe, David Quarcoo, Norman Schöffel, Eileen M. Wanke, Daniela Ohlendorf, Doris Klingelhöfer, David A. Groneberg, Stefanie Mache

**Affiliations:** 1 Department of Gynecology and Obtestrics, Goethe University Frankfurt, Theodor-Stern-Kai 7, 60590 Frankfurt, Germany; 2 Division of Social Medicine, The Institute of Occupational Medicine, Social Medicine and Environmental Medicine, Goethe University Frankfurt, Theodor-Stern-Kai 7, 60590 Frankfurt, Germany; 3 Department of Medicine/Psychosomatics, Charité, Universitätsmedizin Berlin, Free University and Humboldt University, Luisenstrasse 13a, 10117 Berlin, Germany

**Keywords:** Task analysis, Job situation, Compensation, Gynecology - obstetrics

## Abstract

Beginning in the first decade of the 21st centruy, there was a growing disregard for the benefits of the German medical system concerning the junior obstetricians/gynecologists (OB/GYN) job situation. As in other fields of medicine, numerous colleagues left Germany to work in other countries such as the United Kingdom, Noway, Sweden, or Switzerland. According to studies, financial factors represent one of the reasons for the discontent. We here present a practical descriptive approach to assess/review the actual compensation of single work tasks of OB/GYNs on the basis of previously published, existing data. Using the workflow data from the Medical work Assessment in German hospitals (MAGRO) platform of twenty junior OB/GYNs with an average workday of 9:24:35 h (SD = 01:05:07 h), a large scale data analysis of 2,325,556 different time points was performed to calculate the financial valuation of single work tasks. In order to assess the evolution over the past years, different modern and historic (e.g. AiP) pay scales were used and analysed in relation to the actual work on a weekly, monthly and per annum basis. Our review shows that there has been a dramatic increase in the financial reward of the practical work tasks of junior OB/GYN physicians in German hospitals in comparison to the situation of the early 2000s years. In this respect, it can not be further argued that the German system has large disadvantages concerning the payment of junior doctors in comparison to other European countries.

## Introduction

The common occupational health problem attributed to healthcare work is infection [1–9]. But healthcare workers also have a large variety of other job-related risk factors [10–13]. Over the past 20 years, a number of studies reported a growing discontent in job satisfaction among young physicians in the German health care system [14–16]. Similar reports in the field of obstetrics and gynecology were reported in other countries [17–19]. These studies predominantly focused on factors such as occupational stress, satisfaction, and burnout [20, 21]. Parallel to these reports, a high rate of young doctors left their country of origin for foreign work places and a high number of German hospitals currently encounter problems concerning junior residents. This severe problem targets one of the key fundaments of the health service – the pyramidal system of junior residents developing into specialists who then continue to work either as hospital or outpatient consultants.

Due to the magnitude of the problem concerning the shortage of junior staff and the ongoing complain concerning working conditions, the Medical work Assessment in German hospitals (MAGRO) platform was established in 2009 [22] and has focused on a variety of clinical fields including gynecology and obstetrics [23], general surgery [24], pediatrics, psychiatry [25], emergency care [26], or internal medicine [27, 28], so far. One of these previous studies showed hat the working conditions for junior OB/GYNs are by far not as bad as previously hypothesized. In a second step, MAGRO now focuses on economic aspects in order to demonstrate that the increase in job quality is also present in this area.

In this respect, the present descriptive review focused on historic and current payment scales and relates them to the average work schedule of junior resident OB/GYNs. Since there has been a change in the status of young physicians, large differences can be anticipated and therefore, the hypothesis of the present review is that there has been an improvement in the esteem of single OB/GYNs work tasks.

## Materials and methods

### Setting

This descriptive review belongs to the Medical work Assessment in German hospitals (MAGRO) studies [22]. It aims to assess the improvement of payment aspects within the field of OB/GYN over the past years by reviewing/analysing existing MAGRO data. The descriptive analysis is based on the data of a previous MAGRO study that performed a work task analysis of 20 physicians in OB/GYN wards of three Berlin hospitals in order to provide reference data for daily work [23]. In brief, a work flow analysis was conducted with a mobile personal computer [29] and a special OB/GYN task inventory with 221 different medical activities, grouped into 18 categories (e.g. “telephone call” and “talking to a colleague” was grouped into the “conversation” category) [23] (Table 1). The interobserver reliability exceeded 80% and in total, 564:35:56 h were analysed to provide reference data. In this respect, the average workday of the obstetrician-gynecologists was 9:24:35 h (SD = 1:05:05 h) [23]. The longest workday observed was 12:52:02 h, the shortest was 8:02:37 h. Table 1 summarizes the main work-related activity tasks which were then used for further payment salaries [23].

**Table 1:** Average times for main activities performed by obstetrician-gynecologists [10] and [23]

Categories	Average time of activity (hh:mm:ss)	SD (hh:mm:ss)	Percentage
Ward rounds	00:21:48	00:15:19	3.86
Admission	00:05:05	00:06:23	0.90
Conversation	02:41:56	00:33:35	28.68
Medical consultation	00:39:03	00:20:09	6.92
Administrative work	02:09:40	00:30:59	22.97
Working with the patient	00:10:38	00:09:56	1.88
Supervision, social support	00:00:04	00:00:09	0.01
Diagnostics	00:14:55	00:12:48	2.64
Breaks	00:06:02	00:05:13	1.07
Walking	00:42:03	00:14:00	7.45
Operation	01:19:11	00:55:15	14.03
Research	00:00:35	00:01:46	0.10
Teaching	00:09:08	00:20:31	1.62
Medical emergency	00:00:01	00:00:04	0.00
Work disruptions	00:06:03	00:03:14	1.07
Pregnancy care	00:17:57	00:22:19	3.18
Other	00:20:27	00:11:15	3.62

### Salary scales and calculations

In order to analyse differences in the appreciation of physicians work between the situation ten years ago and nowadays, former and current German pay scales were related to the reference values depicted in Table 1. Out of the variety of over ten different pay scales in Germany, five pay scales were chosen which consist of one rate which was paid eight years ago to physicians in their second residential year after having finished the medical exams (Arzt im Praktikum) [30] and four current rates for physicians in the second year after the final medical exams (Table 2). They consist of two governmental (TVöD paid to federally employed physicians and TV-Ärzte (TdL) paid by physicians employed by single German states [31], a rate paid by a private hospital company (Helios) [32] and a rate paid by municipal hospitals (TV-Ärzte (VKA)) [33]). The single salary scale values were then used to calculate the average costs for single tasks per day, per month and per year.

**Table 2:** Pay scales used to calculate compensation for main work tasks

Pay scales	Pre tax salary per month (€)
Arzt im Praktikum (AiP)	1350.57 €
TVöD Bund	3431.28 €
TV-Ärzte (Helios)	3900.00 €
TV-Ärzte (VKA)	3947.67 €
TV-Ärzte (TdL)	4260.59 €

## Results

The calculation of salary percentages for single work tasks of junior hospital OB/GYN doctors in a setting of a total 564:35:56 h work hours led to a wide range of values with regard to the single tarifs and single work tasks which are depicted in Tables 3, 4, 5, 6, and 7. In total, a magnitude of 2,325,556 different time points were related to the different work tasts and then the average financial compensation per work task was calculated for the different payment scales: The lowest values were found in the historic AiP payment scale. In this scale which was used until 2004, the average compensation i.e. for the work task “adminstrative work” (02:09:40 +/− 00:30:59 hh:mm:ss per day) was 14.47 € per day, 313.52 € per month and 3762.28 € per year (Table 3). By contrast, in the payment scale for physicians employed by the federal government the exactly same task is valued 36.76 € per day, 796.54 € per month and 9558.51 € per year (Table 4). In the Helios payment scale, the adminstrative work is compensated with 10,864.23 € per year (Table 5), in the payment scale for municipally employed physicians with 8833.45 € per annum (Table 6) and in the current payment scale of the German states (TdL) which is i.e. paid at the university schools of medicine in some of the 16 German states, this work category is valued 11,868.72 € (Table 7). That means that not longer than eight years prior to the last time point here observed, a physicians work force at this stage of the career was valued less than 1/3 of todays valuation.

**Table 3:** Average compensation per main activity of physician in the AIP payment scale (work experience approx. 2 years)

Categories	Average time main activities	SD time main activities	Average compensation/main activity/day	Average compensation/main activity/month	Average compensation/main activity/year	Percentage of total
	(hh:mm:ss)	(hh:mm:ss)	(€)	(€)	(€)	
Administrative work	02:09:40	00:30:59	14.47	313.52	3762.28	22.97%
Medical consultation	00:39:03	00:20:09	4.36	94.42	1133.04	6.92%
Admission	00:05:05	00:06:23	0.57	12.29	147.49	0.90%
Conversation	02:41:56	00:33:35	18.07	391.54	4698.50	28.68%
Research	00:00:35	00:01:46	0.07	1.41	16.93	0.10%
Diagnostics	00:14:55	00:12:48	1.66	36.07	432.81	2.64%
Teaching	00:09:08	00:20:31	1.02	22.08	265.00	1.62%
Medical emergency	00:00:01	00:00:04	0.00	0.04	0.48	0.00%
Surgery	01:19:11	00:55:15	8.84	191.46	2297.51	14.03%
Work disruptions	00:06:03	00:03:14	0.68	14.63	175.54	1.07%
Pregnancy care	00:17:57	00:22:19	2.00	43.40	520.82	3.18%
Other	06:49:01	00:11:15	2.28	49.45	593.36	3.62%
Supervision. social support	00:00:04	00:00:09	0.01	0.16	1.93	0.01%
Working with the patient	00:10:38	00:09:56	1.19	25.71	308.53	1.88%
Ward round	00:21:48	00:15:19	2.43	52.71	632.53	3.86%
Walking time	00:42:03	00:14:00	4.69	101.67	1220.08	7.45%

**Table 4:** Average compensation per main activity of physician in the TVöD Bund payment scale (work experience approx. 2 years)

Categories	Average time main activities	SD time main activities	Average compensation/main activity/day	Average compensation/main activity/month	Average compensation/main activity/year	Percentage of total
	(hh:mm:ss)	(hh:mm:ss)	(€)	(€)	(€)	
Administrative work	02:09:40	00:30:59	36.76	796.54	9558.51	22.97%
Medical consultation	00:39:03	00:20:09	11.07	239.88	2878.61	6.92%
Admission	00:05:05	00:06:23	1.44	31.23	374.72	0.90%
Conversation	02:41:56	00:33:35	45.91	994.76	11,937.08	28.68%
Research	00:00:35	00:01:46	0.17	3.58	43.00	0.10%
Diagnostics	00:14:55	00:12:48	4.23	91.63	1099.60	2.64%
Teaching	00:09:08	00:20:31	2.59	56.11	673.27	1.62%
Medical emergency	00:00:01	00:00:04	0.00	0.10	1.23	0.00%
Surgery	01:19:11	00:55:15	22.45	486.42	5837.08	14.03%
Work disruptions	00:06:03	00:03:14	1.72	37.17	445.98	1.07%
Pregnancy care	00:17:57	00:22:19	5.09	110.27	1323.20	3.18%
Other	06:49:01	00:11:15	5.80	125.62	1507.49	3.62%
Supervision. social support	00:00:04	00:00:09	0.02	0.41	4.91	0.01%
Working with the patient	00:10:38	00:09:56	3.01	65.32	783.85	1.88%
Ward round	00:21:48	00:15:19	6.18	133.92	1607.01	3.86%
Walking time	00:42:03	00:14:00	11.92	258.31	3099.76	7.45%

**Table 5:** Average compensation per main activity of physician in the TV-Ärzte (Helios) payment scale (work experience approx. 2 years)

Categories	Average time main activities	SD time main activities	Average compensation/main activity/day	Average compensation/main activity/month	Average compensation/main activity/year	Percentage of total
	(hh:mm:ss)	(hh:mm:ss)	(€)	(€)	(€)	
Administrative work	02:09:40	00:30:59	41.79	905.35	10,864.23	22.97%
Medical consultation	00:39:03	00:20:09	12.58	272.65	3271.84	6.92%
Admission	00:05:05	00:06:23	1.64	35.49	425.91	0.90%
Conversation	02:41:56	00:33:35	52.19	1130.64	13,567.72	28.68%
Research	00:00:35	00:01:46	0.19	4.07	48.88	0.10%
Diagnostics	00:14:55	00:12:48	4.81	104.15	1249.80	2.64%
Teaching	00:09:08	00:20:31	2.94	63.77	765.24	1.62%
Medical emergency	00:00:01	00:00:04	0.01	0.12	1.40	0.00%
Surgery	01:19:11	00:55:15	25.52	552.87	6634.44	14.03%
Work disruptions	00:06:03	00:03:14	1.95	42.24	506.90	1.07%
Pregnancy care	00:17:57	00:22:19	5.78	125.33	1503.96	3.18%
Other	06:49:01	00:11:15	6.59	142.78	1713.42	3.62%
Supervision. social support	00:00:04	00:00:09	0.02	0.47	5.59	0.01%
Working with the patient	00:10:38	00:09:56	3.43	74.24	890.92	1.88%
Ward round	00:21:48	00:15:19	7.03	152.21	1826.53	3.86%
Walking time	00:42:03	00:14:00	13.55	293.60	3523.19	7.45%

**Table 6:** Average compensation per main activity of physician in the TV-Ärzte (VKA) payment scale (work experience approx. 2 years)

Categories	Average time main activities	SD time main activities	Average compensation/main activity/day	Average compensation/main activity/month	Average compensation/main activity/year	Percentage of total
	(hh:mm:ss)	(hh:mm:ss)	(€)	(€)	(€)	
Administrative work	02:09:40	00:30:59	42.30	916.42	10,997.02	22.97%
Medical consultation	00:39:03	00:20:09	12.74	275.99	3311.83	6.92%
Admission	00:05:05	00:06:23	1.66	35.93	431.12	0.90%
Conversation	02:41:56	00:33:35	52.82	1144.46	13,733.55	28.68%
Research	00:00:35	00:01:46	0.19	4.12	49.47	0.10%
Diagnostics	00:14:55	00:12:48	4.87	105.42	1265.08	2.64%
Teaching	00:09:08	00:20:31	2.98	64.55	774.60	1.62%
Medical emergency	00:00:01	00:00:04	0.01	0.12	1.41	0.00%
Surgery	01:19:11	00:55:15	25.83	559.63	6715.53	14.03%
Work disruptions	00:06:03	00:03:14	1.97	42.76	513.10	1.07%
Pregnancy care	00:17:57	00:22:19	5.86	126.86	1522.34	3.18%
Other	06:49:01	00:11:15	6.67	144.53	1734.36	3.62%
Supervision. social support	00:00:04	00:00:09	0.02	0.47	5.65	0.01%
Working with the patient	00:10:38	00:09:56	3.47	75.15	901.81	1.88%
Ward round	00:21:48	00:15:19	7.11	154.07	1848.86	3.86%
Walking time	00:42:03	00:14:00	13.72	297.19	3566.26	7.45%

**Table 7:** Average compensation per main activity of physician in the TV-Ärzte (TdL) payment scale (work experience approx. 2 years)

Categories	Average time main activities	SD time main activities	Average compensation/main activity/day	Average compensation/main activity/month	Average compensation/main activity/year	Percentage of total
	(hh:mm:ss)	(hh:mm:ss)	(€)	(€)	(€)	
Administrative work	02:09:40	00:30:59	45.65	989.06	11,868.72	22.97%
Medical consultation	00:39:03	00:20:09	13.75	297.86	3574.35	6.92%
Admission	00:05:05	00:06:23	1.79	38.77	465.29	0.90%
Conversation	02:41:56	00:33:35	57.01	1235.18	14,822.17	28.68%
Research	00:00:35	00:01:46	0.21	4.45	53.39	0.10%
Diagnostics	00:14:55	00:12:48	5.25	113.78	1365.36	2.64%
Teaching	00:09:08	00:20:31	3.22	69.67	836.00	1.62%
Medical emergency	00:00:01	00:00:04	0.01	0.13	1.53	0.00%
Surgery	01:19:11	00:55:15	27.88	603.99	7247.85	14.03%
Work disruptions	00:06:03	00:03:14	2.13	46.15	553.77	1.07%
Pregnancy care	00:17:57	00:22:19	6.32	136.92	1643.01	3.18%
Other	06:49:01	00:11:15	7.20	155.99	1871.84	3.62%
Supervision. social support	00:00:04	00:00:09	0.02	0.51	6.10	0.01%
Working with the patient	00:10:38	00:09:56	3.74	81.11	973.30	1.88%
Ward round	00:21:48	00:15:19	7.67	166.28	1995.41	3.86%
Walking time	00:42:03	00:14:00	14.80	320.75	3848.94	7.45%

The leading work task concerning the time is the conversation with colleagues and staff members. This time period of 2:41:56 +/− 0:33:35 h was compensated with 18.07 € per day in the AiP scale (391.54 € per month and 4698.50 € per year, Tab. 3), 45.91 € per day in the TVöD Bund payment scale (994.76 € per month and 11,937.08 € per year, Tab. 4), 52.19 € per day in the TV Helios (1130.64 € per month and 13,567.72 € per year, Tab. 5), 52.82 € per day in the TV-Ärzte (VKA) payment scale (1144.46 € per month and 13,733.55 € per year, Tab. 6), and 57,01 € per day in the TV-Ärzte (TdL) payment scale (1235.18 € per month and 14,822.17 per year, Tab. 7).

**Fig. 1:**
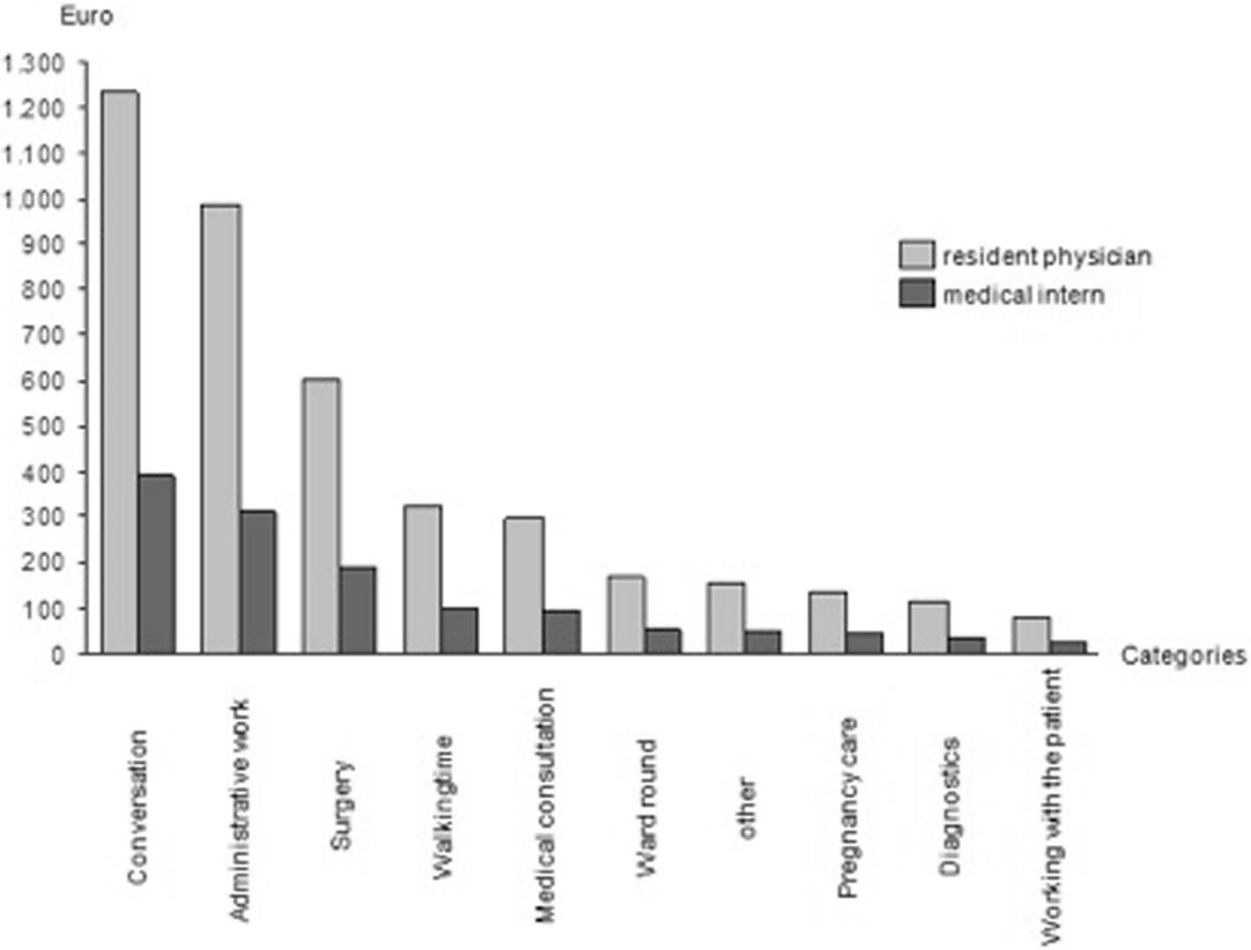
Average labor costs of the most frequently main activities/month. Comparison between the historic AiP scale and the current TdL scale

When comparing the different payment scales, i.e. by focusing on the lowest payment scale and the highest (Fig. 1), large differences are found. I.e. when analysing the pure walking time of 42 +/− 14 min per day, it can be seen that this category is currently valued 320.75 € per month (3848.94 € per year) in the TdL scale. This is even higher than the amount of money directed to adminstrative work (over 2 +/− 0.5 h/d) in the historic AiP scale (313.52 € per month/ 3762.28 € per year) which was paid for doctors of the same qualification (2 years of experience) until 2004 (Fig. 1).

## Discussion

Among the numerous occupational problems found in health care personel [34–42], there are many studies that address career satisfaction and perceptions of quality among obstetricians and gynecologists [17, 19, 43–46]. The present article does not want to add a further questionnairy-based survey which asks physicians for their attitude concerning job situation, payment or other factors but addresses a difficult area – namely the field of young academics in the field of obstetrics and gynecology – by reviewing existing real-time analysis data and correlating this data to tarfis. After having previously recorded reference values for daily work activities of junior OB/GYN physicians [23], the aim of this MAGRO review study was to analyse the valuation for the different work activities in relation to pay scales which were paid in a 8 year intervall to physicians in their second year after the final medical exams. In this respect, it is important to notice that the reference average workday of the OB/GYNs which was identified on the basis of an analysis of more than 2 million time points was found not to be longer than 9:24:35 h (SD = 1:05:05 h). This is shorter than anticipated and reported in other studies [47] and from these data, it cannot be stated that the average junior physician works more that 10–12 h per workday. Also, this amount of daily work time leads to a higher proportion of money allocated to single work tasks, as presently calculated. In this respect, we here report that i.e. the amount of salary allocated to the most frequent work task – conversation – of a physician two years after the final medical exams i.e. in the highest payment scale (TV-Ärzte TdL) is 1235.18 € per month (14,822.17 per year). This amount is more than three-fold higher than the amount allocated in the AiP payment scale (391.54 € per month and 4698.50 € per year). It can therefore be clearly stated that there is a significant increase of the esteem of the workforce of a junior physician on a monetary basis. This increase in the compensation dramatically differs to the public opinion of the current job situation of young doctors in Germany in 2007 [20]. This can also be concluded by comparing the reward for different work tasks. I.e. the pure walking time is rewared in current pay scales as high as a much more time-consuming work tasks, i.e. administrative work in the historic AiP pay scale. By comparing the exact values, it can be estimated that the increase in financial estimation is about 3-fold. In contrast to reports that state a difficult work situation for hospital doctors, the present study at least demonstrates a strong increase in the financial appreciation.

### Limitations

The key message of the present review of data is that single work tasks and overall work of young German physicians working in OB/GYN are presently valued about 300% higher than in the AiP pay scale. This increase in estimation influences all aspects of work life. However, the present study harbors several limitations that need to be adressed. First, our data basis refers to an average daily working time of 9:24:35 h (SD = 1:05:05 h) which is lower than anticipated in many studies which address the occupational situation of German physicians. Although our data calcuation is based on the analysis of over 2,000,000 time points, it needs to be noted that they are of cause specific to hospital size and geographical location and should therefore be interpreted with caution. As discussed earlier, a further limitation of the MAGRO studies is that they so far only observe work that takes place on day shifts of weekdays and do not include information about medical workflow of on-call weekend and night shifts. Future studies may be extended to include these time periods in order to obtain more detailed information.

A further limitation of the MAGRO studies is that the category of work activities was not chosen systematically. Therefore, the currently used categorization may lead to measurement bias, particularly in some categories including conversation, medical consultation, social works, and others may overlap and could lead to over measurement.

Concerning the different pay scales that were used to calculate the valuation of work, it needs to be stated that they change with time. However, we tried to identify tariffs which are used in a large proportion of hospitals, i.e. municpal, private and state-owned hospitals.

For the future, there is also a comparison analysis with senior OB/GYN doctors needed. This should adress average times for main activities and scale of salary. Due to differences of the level of professional experience, skills and duties, major differences concerning compensation and payment can be anticipatetd.

## Conclusion

This is the first review that combines the data of a realtime workflow analysis with more than 2,000,000 time points with pay scale data in order to assess the merit of junior OB/GYN work tasks. With regard to the numerous scales that were used, it can be stated that there is an increase in the valuation of the work force of junior OB/GYN doctors of about 300%. In contrast to the multitude of studies that report stressful conditions within the daily routine, the present study adds a new context to the discussion and demonstrates that at least, the work is now compensated in a more appropriate way.
